# Supplementary material for: Demographic, ecological, and physiological responses of ringed seals to an abrupt decline in sea ice availability
Source: PeerJ. 2017 Feb 2;5:e2957. doi: 10.7717/peerj.2957 (PMC5292026; doi:10.7717/peerj.2957)
Supplement: Supplemental Information 4 — North Atlantic Oscillation (NAO), Atlantic Oscillation (AO), El Nino Southern Oscillation (ENSO), breakup and freeze-up dates. [file peerj-05-2957-s004.docx]

Supplementary Table1. 1971-2014 NAO, AO, ENSO, and eastern Hudson Bay breakup and freeze-up from 1979 to 2014.

| Year | AO | ENSO | NAO | Breakup | Freeze-up |
| --- | --- | --- | --- | --- | --- |
| 1971 | -0.49459 | -1.3 | -0.69667 |  |  |
| 1972 | 0.264983 | -0.7 | 0.396667 |  |  |
| 1973 | 1.08517 | 1.7 | 0.36 |  |  |
| 1974 | -0.1462 | -1.7 | 0.506667 |  |  |
| 1975 | 0.781803 | -0.5 | 0.486667 |  |  |
| 1976 | 0.993478 | -1.5 | 0.226667 |  |  |
| 1977 | -2.6173 | 0.7 | -1.04333 |  |  |
| 1978 | -1.20007 | 0.7 | -0.84667 |  |  |
| 1979 | -1.30322 | 0 | -1.20667 | 166 | 340 |
| 1980 | -0.56821 | 0.6 | 0.1 | 163 | 335 |
| 1981 | -0.16841 | -0.2 | 0.69 | 191 | 337 |
| 1982 | -0.37507 | 0 | 0.08 | 164 | 334 |
| 1983 | 0.17346 | 2.1 | 0.946667 | 171 | 339 |
| 1984 | 0.262857 | -0.5 | 0.89 | 156 | 328 |
| 1985 | -1.2665 | -0.9 | -0.7 | 151 | 333 |
| 1986 | -1.80645 | -0.4 | 0.11 | 185 | 326 |
| 1987 | -0.85368 | 1.1 | -0.29667 | 180 | 345 |
| 1988 | -0.44515 | 0.8 | 0.7 | 154 | 342 |
| 1989 | 2.688033 | -1.6 | 1.26 | 180 | 334 |
| 1990 | 1.252883 | 0.1 | 0.433333 | 180 | 335 |
| 1991 | 0.374647 | 0.4 | 0.706667 | 174 | 336 |
| 1992 | 1.094977 | 1.6 | 0.466667 | 181 | 329 |
| 1993 | 1.768833 | 0.2 | 0.856667 | 169 | 328 |
| 1994 | -0.41784 | 0.1 | 1.02 | 180 | 348 |
| 1995 | 0.722987 | 0.9 | 1.363333 | 173 | 334 |
| 1996 | -1.05476 | -0.9 | -0.62 | 184 | 357 |
| 1997 | -0.09629 | -0.5 | -0.06667 | 164 | 330 |
| 1998 | -0.7783 | 2.1 | -0.22667 | 159 | 352 |
| 1999 | 0.648627 | -1.4 | 0.643333 | 146 | 350 |
| 2000 | 1.1297 | -1.6 | 1.303333 | 157 | 351 |
| 2001 | -1.31188 | -0.7 | 0.04 | 150 | 361 |
| 2002 | 0.454133 | -0.2 | 0.236667 | 171 | 338 |
| 2003 | -0.64532 | 0.9 | -0.05333 | 185 | 362 |
| 2004 | -0.98303 | 0.3 | 0.07 | 177 | 338 |
| 2005 | 0.105223 | 0.6 | 0.89 | 158 | 350 |
| 2006 | -0.81005 | -0.7 | 0.104837 | 145 | 355 |
| 2007 | 1.002867 | 0.7 | 0.36307 | 173 | 345 |
| 2008 | 0.859387 | -1.4 | 0.6561 | 150 | 348 |
| 2009 | 0.25841 | -0.8 | -0.07584 | 184 | 347 |
| 2010 | -3.42177 | 1.3 | -1.67293 | 141 | 369 |
| 2011 | -0.9129 | -1.3 | -0.67427 | 163 | 349 |
| 2012 | 0.654935 | -0.7 | 1.371767 | 159 | 348 |
| 2013 | -1.12184 | -0.4 | 0.02096 | 159 | 346 |
| 2014 | 0.183305 | -0.5 | 0.85704 | 168 | 341 |
