# Supplementary material for: Demographic, ecological, and physiological responses of ringed seals to an abrupt decline in sea ice availability
Source: PeerJ. 2017 Feb 2;5:e2957. doi: 10.7717/peerj.2957 (PMC5292026; doi:10.7717/peerj.2957)
Supplement: Supplemental Information 5 — Date, Comment, Reporter. [file peerj-05-2957-s005.docx]

Supplementary Table2. Chronology of unusual ringed seal and polar bear observations gathered from Hudson Bay communities related to a warming event in 2010.

| Date | Comment | Reporter |
| --- | --- | --- |
| 4 Nov. 2010 | I've only seen about 3 kills in the 11 years I've worked for you guys and now 7 in a month? | Marc Hebert, Manitoba Conservation Officer |
| 14 Nov. 2010 | He has also seen quite a few seals and seal kills by Polar Bears. He also flew over Button Bay and saw a number of seal kills that hadn't been consumed. | Mike Macri (Sea North Tours, Churchill) |
| 16 Nov. 2010 | He states that seals are venturing inland than normal. Bears are eating seals. The only physical problems or abnormalities he notes he has seen is one seal that appeared to be bleeding from the anus. Sick seals -- showing evidence of hair loss. | Amanda Currie (DFO) conversation with Donnie (Great White Bear tours). |
| 16 Nov. 2010 | Recently found a seal that was still alive but crawling over land near the Rx road just out of the Town of Churchill. | LeeAnn Fishback (CNSC) with Manitoba Conservation |
| 17 Nov. 2010 | First Vince had heard of dead seals. But noted Darryl Hedman flew coast and saw over 300 bears - saw 18 dead seals that had been killed so he says by bears - when I asked him how the bears were catching seals he said they are likely getting caught on the flats when tide goes out and bears just taking advantage of easy meal - maybe something wrong with seals that they are getting caught like this. | Ole Nielson (DFO Science) with Vince Crichton (Manitoba Conservation Manager, Game Fur & Problem Wildlife) |
| 18 Nov. 2010 | They both confirm they’re have been no reports of any killer whale sightings in the area, as its too late in the seasons for Killer Whales. Also the local polar bears are also very fat, and several appear to be 'stock piling' the seals they catch (i.e. some people have witness and photographed the bears stock piling or buried seals inland instead of eating them). Mike was on a flight a week ago and saw a fat polar bear kill a seal, walk away and kill another seal on the shore, drag it back to the first, and then walk away without eating either. And another sow with cubs had a dead seal and was not eating it. Another seal was seen moving along RX road about 1-2 km from shore. Received two pictures of this from Mike Macri. | Tara Bortoluzzi (DFO Science) spoke with Mike Macri and Bob Windsor (Conservation Officer in Churchill) |
| 24 Nov. 2010 | I met two hunters from Chesterfield Inlet and Whale Cove in the Iqaluit airport on Monday that were also very concerned with the 'behavior' of ringed seals near their communities this fall. I'm following up with them and several other HTOs. They reported that they are catching more adult seals this year which are really large, and very few pups. The seals are also very easy to catch, in many cases they said 'too easy'. One hunter caught 30 seals in one day trip. The seals are also coming inland and hanging around the shoreline for extended periods of time. Of course, it's great for hunting, but they were really concerned as this is very unusual. | Ole Nielsen (DFO) |
| 26 Nov. 2010 | Some of the Hunters and Trappers Organizations in the Kivalliq region have recently reported concerns with ‘odd behaviour’ of ringed seals near the communities (i.e. seals coming close to shore and hanging around, and hauling out on shore), as well as some seals that appears to be sick (i.e. molting and loss of hair, seal pocks, low fat content, etc.). | Tara Bortoluzzi (DFO) summarizing response from Kivalliq Region communities |
| 26 Nov. 2010 | She has heard the same concerns from hunters: “After discussion with my board of directors, they have reported some hunters catching seals on shore, and far away from shore with loss of hair (like bald patches) but nobody took pictures and the seals were used as dog food.” | Leah Muckpah (Arviat HTO Manager) |
| 27 Nov. 2010 | He hasn't seen any more seals on shore, neither have the helicopters or tundra buggy camps. Also notes another odd thing, the zodiacs, for the first time ever, are covered with scratches from bearded seals who were hauling up into them in September. | email from Mike Macri (Sea North Tours) |
| 17 Dec. 2010 | “A couple weeks ago while he was out of town, Lucasssie Takatak, found 6 dead seals on the beach, their heads seem to be craving for air like their heads up back.” | Lucassie Arragutainaq (Secretary Manager Sanikiluaq HTO) |
| 06 Jan. 2011 | They observed a very few number of seals were shedding and that even a few number of them were sinking after being shot. The seal harvest in Arviat is usually done when the first ice forms in the salt water, usually late October to Late November. During that time, it is unheard of that seals would be shedding fur and that they would sink after being shot. | reports from local hunters of Arviat |
| 26 Jan. 2011 | Ringed seals usually molt in the spring but locals noted seals molting in the fall. The local Conservation Officer sent seal parts to DFO showing the unusual molt. During fall large numbers (100s) of seals were observed along shorelines. Other communities including Repulse Bay also noted the same unusual conditions. Coral Harbour seldom sees seals near town but this past fall large numbers were in the Harbour and some went on land in the harbour (very unusual).  His own personal experience - he was traveling along shoreline in August and found a seal on the beach. The ringed seal kept traveling up the shore – unusual behaviour. Three weeks later he was in a different area and saw a harp seal on land – about ¼ mile inland. It was a late freeze up this autumn and a very warm fall. The ice formed along the shoreline twice in mid-November but drifted off with winds both times before it finally formed fast in December. In December rain fell. | Ferguson phone conversation with Noah Nakoolak of Coral Harbour HTO |
